# ﻿*Oblatopyrochroabellula*, an enigmatic new genus and species of Pyrochroinae (Coleoptera, Pyrochroidae) from Xizang, China

**DOI:** 10.3897/zookeys.1191.118653

**Published:** 2024-02-16

**Authors:** Qi Gao, Daniel K. Young, Zhao Pan

**Affiliations:** 1 Key Laboratory of Zoological Systematics and Application, School of Life Sciences, Hebei University, 071002, Baoding, Hebei Province, China Hebei University Baoding China; 2 Department of Entomology, University of Wisconsin, Madison, WI 53706, USA University of Wisconsin Madison United States of America

**Keywords:** Fire-colored beetle, taxonomy, Tibet

## Abstract

*Oblatopyrochroabellula*, a new genus and species of Pyrochroinae Latreille, 1807 from Xizang, China, is described and illustrated. The antennae, cranial apparatus, and genitalia of the new genus form a truly unique set of characters not observed in any other pyrochroid genus. The taxonomic position and phylogenetic relationships of *Oblatopyrochroa***gen. nov.** are also discussed but appear difficult to resolve.

## ﻿Introduction

Pyrochroinae Latreille, 1807 is the most speciose subfamily of Pyrochroidae Latreille, 1807 and is widely distributed in the Holarctic Region, especially in temperate areas of Asia. This subfamily includes more than 120 recent species in 14 genera (Young 1975, 2002; Young et al. 2020; Pan et al. 2021; Molfini et al. 2022): *Dendroides* Latreille, 1810 (8 species), *Dendroidopsis* Young, 2004b (4 species), *Eupyrochroa* Blair, 1914 (1 species), *Frontodendroidopsis* Young, 2004b (3 species), *Hemidendroides* Ferrari, 1869 (4 species), *Himalapyrochroa* Young, 2004a (2 species), *Neopyrochroa* Blair, 1914 (4 species), *Phyllocladus* Blair, 1914 (5 species), *Pseudodendroides* Blair, 1914 (7 species), *Pseudopyrochroa* Pic, 1906 (ca 70 species), *Pyrochroa* Geoffroy, 1762 (5 species), *Pyroghatsiana* Young, 2016 (1 species), *Schizotus* Newman, 1838 (5 species), and *Sinodendroides* Young, 2005 (2 species). However, most of the abovementioned genera include only few species, the only world revision was admittedly preliminary (Blair 1914), and no comprehensive taxonomic revision including phylogenetic hypotheses has been published for this subfamily. The taxonomic validity of some genera has been debated (e.g. *Eupyrochroa*, *Pseudopyrochroa*, etc.; Gao et al. 2024).

In May 2023, a unique species of fire-colored beetle was discovered in Xizang, China. The antennae, cranial apparatus, and male genitalia form a truly unique set of characters not observed in any other described pyrochroid genus. Therefore, we propose it as a new pyrochroine genus, which is described and illustrated below.

## ﻿Material and methods

The male holotype is deposited at the Museum of Hebei University, Baoding, China (**MHBU**). The specimen was studied using a Nikon SMZ1500, and the images were taken using a Canon EOS 5D Mark III (Canon Inc., Tokyo, Japan) with a Laowa FF 100 mm F2.8 CA-Dreamer Macro 2× or Laowa FF 25 mm F2.8 Ultra Macro 2.5–5× (Anhui Changgeng Optics Technology Co., Ltd, Hefei, China). The figure of the antenna was drawn by hand using a Nikon SMZ1500 with a camera lucida. Label data are presented verbatim. Line breaks on labels are denoted by a double slash (//); metadata and notes (not written on the labels, themselves) are presented in square brackets ([]). Scientific names are uniformly presented in italics.

Most of the terms in the description are from previous literature (e.g. Young 1975). The ocular index (OI) was first used to quantify the relative distance between the compound eyes of Alleculinae Laporte, 1840 (Campbell and Marshall 1964).

$\mathrm{OI}=\frac{\mathrm{Minimum}\;\mathrm{dorsal}\;\mathrm{distance}\;\mathrm{between}\;\mathrm{compound}\;\mathrm{eyes}\;}{\mathrm{Maximal}\;\mathrm{dorsal}\;\mathrm{width}\;\mathrm{across}\;\mathrm{compound}\;\mathrm{eyes}\;}\times 100$.

## ﻿Results

### ﻿ Oblatopyrochroa

Taxon classificationAnimaliaColeopteraPyrochroidae

Gao, Young & Pan gen. nov.

19E20170-D33F-57BF-A8A5-7140FE786B6C

https://zoobank.org/87AF31B8-8653-45FB-801C-3F9A4E1154B3

Fig. 1

#### Type species.

*Oblatopyrochroabellula*, new species, by monotypy and present designation.

#### Diagnosis.

**Figure 1.**
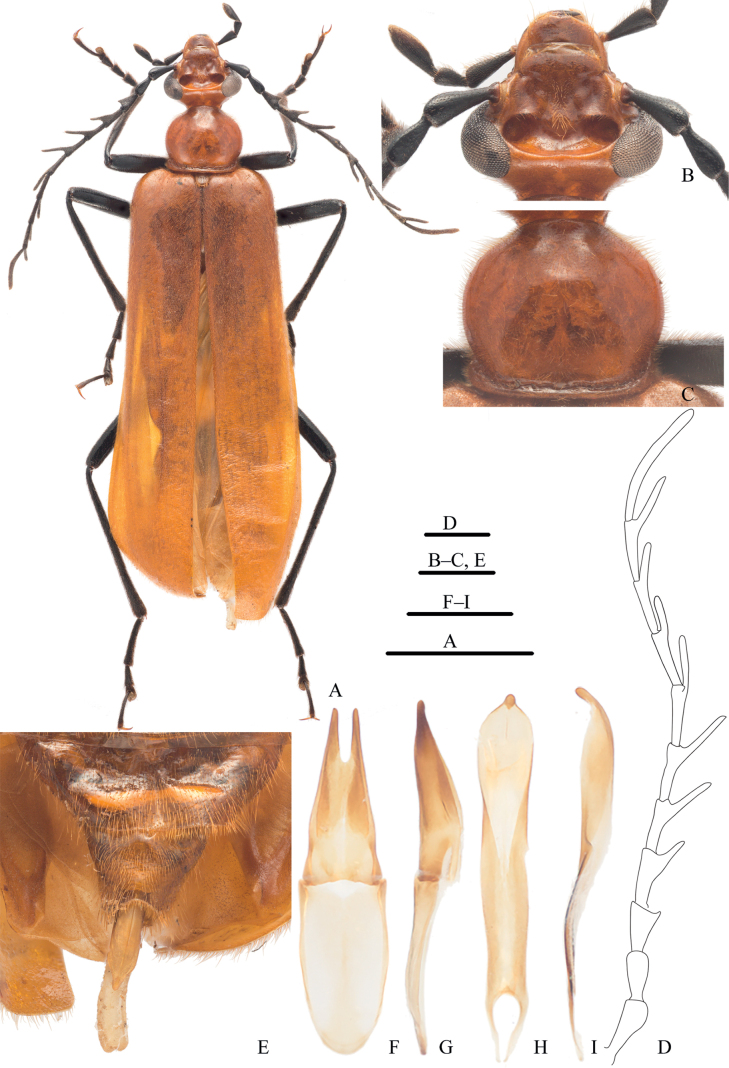
*Oblatopyrochroabellula*, gen. et sp. nov., male, holotype **A** habitus, dorsal view **B** head, dorsal view **C** pronotum, dorsal view **D** antenna, left **E** abdominal sternites VII–VIII, ventral view **F, G** tegmen: **F** dorsal view **G** lateral view **H, I** penis: **H** dorsal view **I** lateral view. Scale bars: 5 mm (**A**); 1 mm (others).

This new genus is easily distinguished from other pyrochroine genera by the combination of following characters: frons with a single, large transverse concavity between compound eyes (Fig. 1B); eyes of moderate size, transverse width of an eye less than width between eyes, dorsally (Fig. 1B); antennal pedicel long, approximately 0.8× length of scape, dorsal face slightly concave (Fig. 1D); parameres of a male genitalia fused along approximately basal 2/3 and lacking recurved apical hooks (Fig. 1F, G).

#### Description.

**Male: *head*** (Fig. 1B) subtriangular, widest at level of eyes, abruptly constricted behind compound eyes, forming conspicuous “neck”. Temples strongly reduced, not prominent. Frons with a large, transverse concavity, flat anteriad cranial excavation, between antennal insertions. Eyes of moderate size, separated dorsally by more than dorsal width of an eye, narrowly separated ventrally. Clypeus flat, frontoclypeal suture not obvious; labrum subsemicircular; mandibular apices acutely bidentate; maxillary cardo well developed, articulating distally with subrectangular basistipes; galea apically fan-shaped, surface with dense, brush-shaped, yellow setae; maxillary palpi 4-segmented, palpomere I shortest, followed by III, II, and IV of similar length, IV longest, subcultriform; labial palpi 3-segmented, I longest, II and III subequal in length, approximately half that of I; mentum rectangular; gula narrow, posterior tentorial pits conspicuous. ***Antennae*** (Fig. 1D) pectinate beyond antennomere III; scape widened at apex; pedicel approximately 0.8× length of scape; flagellomere I serrate, remaining flagellomeres pectinate with rami cylindrical, less than to approximately as long as respective flagellomere, itself.

***Pronotum*** (Fig. 1C) subcircular, approximately as long as wide; disc shining. Prosternum transverse, with transverse rugosities, middle of posterior margin sharply protruding; prothoracic coxal cavities completely open externally and internally. Mesothorax with scutellum small, widest at base, shield-shaped, slightly longer than wide; mesothoracic coxal cavities not closed outwardly by sterna; mesosternum with posterior margin slightly acuminate mesally. Metathoracic coxal cavities closed externally and internally. Elytra elongate, covering abdomen, longitudinal elytral costae obsolete (Fig. 1A). Legs ambulatorial; tarsal formula 5-5-4; each penultimate tarsomere bilobed; pretarsal claws simple.

Abdominal tergites I–II absent, III–VI poorly sclerotized, VII–VIII lightly sclerotized; sternites I–II absent, III–VI with posterior margins nearly parallel, VIII widest basally, apex acutely emarginate mesally (Fig. 1E). Apex of parameres acuminate, without recurved hooks, basal 2/3 fused, dorsal and terminal parts with scattered yellow setae (Fig. 1F, G); penis broadly flattened, median struts paired, short, narrow, wisest subapically, narrowly nodular at apex (Fig. 1H, I).

#### Etymology.

From the Latin root “*oblat*-” for “spread out” and *Pyrochroa*, in reference to the single, large transverse concavity on the frons, putatively diagnostic for the genus. This generic name is feminine.

#### Distribution.

China: SE Xizang.

### ﻿ Oblatopyrochroa bellula

Taxon classificationAnimaliaColeopteraPyrochroidae

Gao, Young & Pan sp. nov.

96429AC8-F3A5-5DD6-830E-D4B428C99DF2

https://zoobank.org/38C0977F-0DB3-4847-9E1D-1DFD7059E068

Fig. 1

#### Diagnosis.

This species, the only known member of *Oblatopyrochroa*, can be recognized by the generic diagnosis given above.

#### Description.

**Male: *body*** (Fig. 1A) orange-yellow, except labial palpi, antennae, and legs black; mandibular apices dark brown. Body densely covered with short, fine, orange-yellow setae; dorsal surface of head sparsely setose except for patch of moderately long, mostly retrorse setae along meson of frons and along anterior rim of cranial excavation. Body length: 19.8 mm; humeral width: 4.8 mm.

***Head*** (Fig. 1B) with dense, small punctures, diameter of punctures less than spacing of punctures, each inside with a very fine, medium-length seta. Dorsal distance between compound eyes wide (OI = 49.4). Clypeus and labrum flattened; labrum with anterior margin slightly emarginate. Frons with widely U-shaped, sub-reniform concavity, shallowly excavate mesally and more deeply so on sides near compound eyes, posterior margin complete, anterior margin obsolete mesally. ***Antennae*** (Fig. 1D) extending back to near middle of elytra; flagellomere I shortest; II–IV subequal in length, approximately as long as pedicel.

***Pronotum*** (Fig. 1C) widest in middle, approximately as wide as head, length 0.87× width; disc shining, densely covered with small punctures, each side of base with inconspicuous protuberance; basal marginal bead complete. Scutellar shield densely covered with small punctures. Leg slender; prothoracic tarsomere V longest, I second longest, II–IV gradually shorter; mesothoracic tarsomere I subequal in length to V, II–IV gradually shorter; metathoracic tarsomere I longest, IV second longest, II–III gradually shorter.

Posterior margins of abdominal sternites III–VII subparallel, VIII with posterior margin shallowly, acutely emarginate mesally (Fig. 1E). In dorsal view, parameres subequal in length to phallobase, basal 2/3 of parameres fused (Fig. 1F). Penis broadly flattened, proximal part abruptly narrowed toward apex, apex nodular-shaped and curved ventrally (Fig. 1H, I).

#### Type material.

***Holotype***: ♂, with the following labels: “2023.V.16 // 西藏派墨公路42 km [China, Xizang, Pai Town-Mêdog County Highway 42 km] // 季权宇采 [Quan-Yu Ji leg.] // 河北大学博物馆 [Museum of Hebei University]”, “29.358986°N // 95.134955°E // elev. 1991 m // 河北大学博物馆 [Museum of Hebei University]”, “HOLOTYPE // *Oblatopyrochroabellula* n. sp. // Det. Gao, Young & Pan” (MHBU).

#### Etymology.

The specific epithet comes from the Latin adjective root “*bellula*-” meaning “pretty” or “elegant”, in reference to the beauty of the species.

#### Collecting habitat.

**Figure 2.**
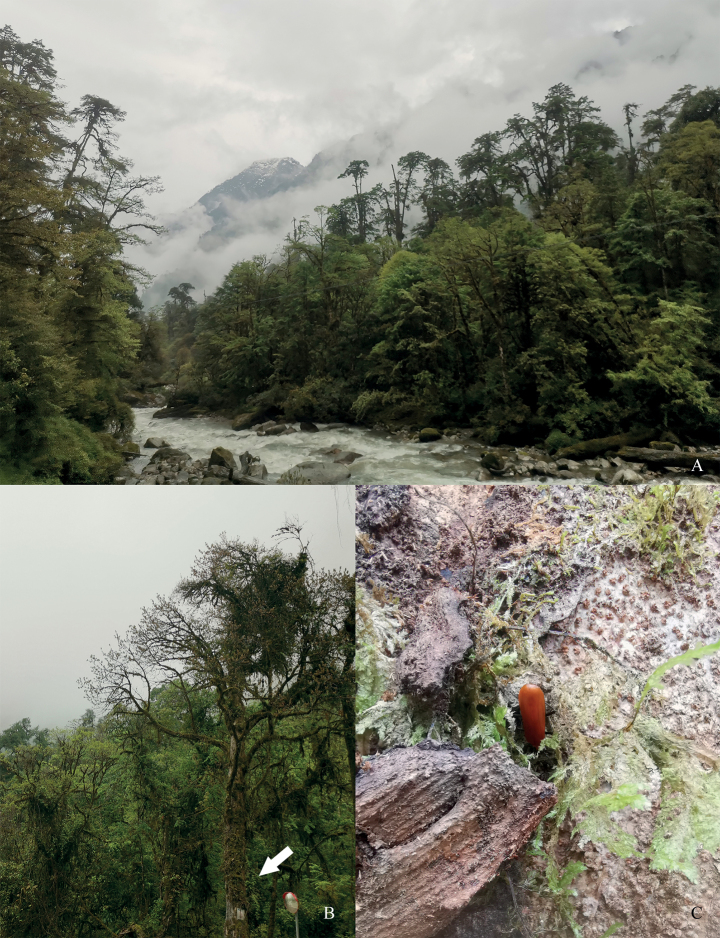
Habitat of *Oblatopyrochroabellula***A** general habitat **B** specific site (indicated by arrow) **C** microhabitat (the beetle inside is the holotype of *O.bellula*). Photographed from China, Xizang, Pai Town-Mêdog County Highway 42 km, elev. 1991 m (type locality), by Quan-Yu Ji.

The holotype was collected in May on the side of a stretch of road from Pai Town to Mêdog County, at a relative low elevation compared to the average elevation in Xizang, with cool temperatures but moist air (Fig. 2A). Specifically, it was found on a dying tree, parts of which had decayed and died (Fig. 2B, C).

#### Distribution.

China: SE Xizang.

## ﻿Discussion

*Oblatopyrochroa* differs from all known pyrochroine taxa and shows a mixed distribution of character states. The three most diagnostic characters of *Oblatopyrochroa* are the shape of the male antennal pedicel, the configuration of the male cranial apparatus (sensu Young 2004b), and the male genitalia. The antennal pedicel of male pyrochroines is subject to significant variation at the generic and specific levels. In *Oblatopyrochroa*, it is elongate and approximately 0.8× the length of the scape, although its shape approached slightly in *Phyllocladuskasantsevi* Young, 2005 (see Young 2013: fig. 13) and *Frontodendroidopsisgibbiceps* Young, 2006.

**Figure 3.**
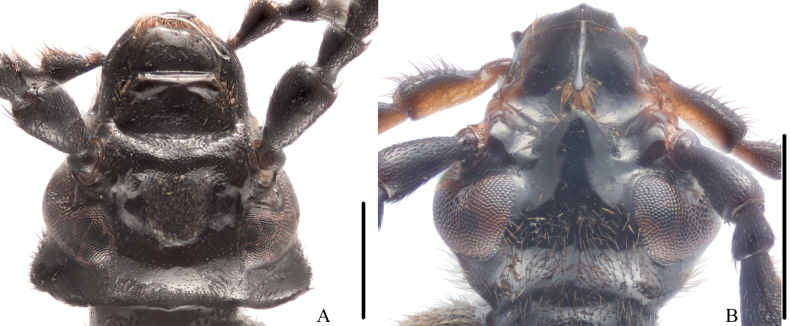
Head of two pyrochroine species, males, dorsal view **A***Eupyrochroainsignita* (Fairmaire, 1894) **B***Pseudopyrochroacarinifrons* Kôno, 1929. Scale bars: 1 mm.

The head of male pyrochroines usually bears one or two pits or depressions that represent important diagnostic features of genera and species. Like several genera and species, *Oblatopyrochroa* has only one cranial pit. However, it is distinctly different from the modifications in *Neopyrochroa* (see Young and Caterino 2007: figs 1C, 2, 3a, 4) and *Phyllocladus* (see Young 2013: figs 2, 3, 8, 9, 12, 14, 15). In genera with a single cranial pit, for example *Eupyrochroa* (Fig. 3A) and *Pyrochroa*, the temples are prominent and the pit is quite shallow.

**Figure 4.**
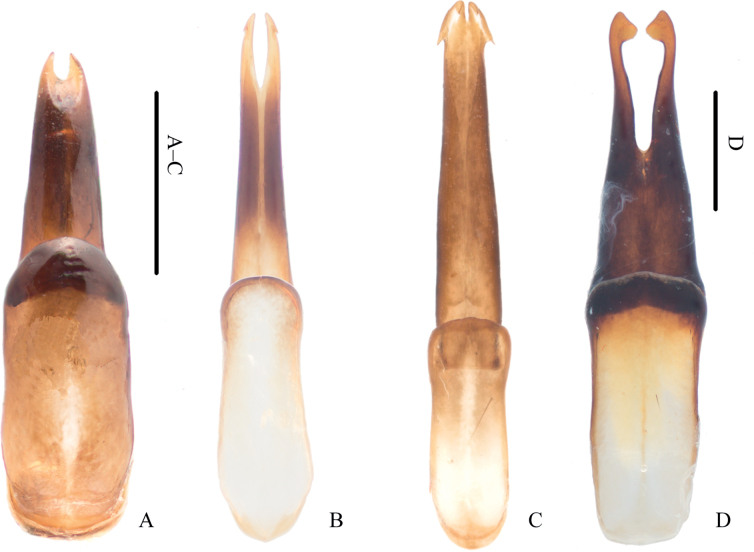
Tegmen of pyrochroine species, dorsal view **A***Eupyrochroainsignita* (Fairmaire, 1894) **B***Frontodendroidopsispennyi* Young, 2017 **C***Pseudopyrochroacarinifrons* Kôno, 1929 **D***Phyllocladusgrandipennis* (Pic, 1906). Scale bars: 1 mm.

The male genitalia of Pyrochroinae have the parameres fused for most of their length, for example *Dendroidopsis*, *Eupyrochroa* (Fig. 4A), *Pseudopyrochroa* (Fig. 4C), *Pyrochroa*, and *Schizotus*, or fused only along the basal half, for example *Himalapyrochroa* and *Phyllocladus* (Fig. 4D). In comparison, the parameres of *Oblatopyrochroa* are fused along the basal 2/3, close to those of *Frontodendroidopsis* (Fig. 4B) but without apically recurved hooks or teeth. The structure of the parameres in *Oblatopyrochroa* is most similar to that of *Eupyrochroa* and *Pyrochroa*. The shape of the penis of *Oblatopyrochroa*, conspicuously widening distally then abruptly “nodular” apically, differs significantly from that of other genera.

Flagellar rami III–IX of *Oblatopyrochroa* are short and thin, similar to *Himalapyrochroa* (Gao et al. 2023; Young 2004a, 2005), not long and almost thread-like, as in most males of *Dendroides*, *Frontodendroidopsis*, and *Sinodendroides*.

The temples of *Oblatopyrochroa* are strongly reduced and not prominent, similar to some species of *Dendroidopsis*, *Frontodendroidopsis*, *Neopyrochroa*, and *Pseudopyrochroa* (Fig. 3B). However, *Dendroidopsis* and *Frontodendroidopsis* usually have large eyes, and the distance between eyes is distinctly less than the dorsal transverse width of each eye in males, which differs from *Oblatopyrochroa*.

Although the observations above clearly support generic recognition, the relationship between the new genus and other pyrochroine genera remains difficult to determine at this time. We anticipate that the relationships will become better resolved by the discovery of larvae and females of *O.bellula*. Additional specimens, together with more material of other genera and species, will enable a more robust molecular phylogenetic analysis of Pyrochroinae as well.

## Supplementary Material

XML Treatment for Oblatopyrochroa

XML Treatment for Oblatopyrochroa bellula

## ﻿Additional information

### Author ORCIDs

Qi Gao https://orcid.org/0000-0002-5902-9276

Daniel K. Young https://orcid.org/0000-0002-5711-2519

Zhao Pan https://orcid.org/0000-0001-7798-0009

### Data availability

All of the data that support the findings of this study are available in the main text.
